# Facile Production Method of PbS Nanoparticles via Mechanical Milling of Galena Ore

**DOI:** 10.3390/mi14030564

**Published:** 2023-02-27

**Authors:** Bety S. Al-Saqarat, Ahmed Al-Mobydeen, Ahmed N. AL-Masri, Muayad Esaifan, Imad Hamadneh, Iessa Sabbe Moosa, Ehab AlShamaileh

**Affiliations:** 1 Department of Geology, The University of Jordan, Amman 11942, Jordan; 2 Department of Chemistry, Faculty of Science, Jerash University, Jerash 26150, Jordan; 3 Department of Studies, Research and Development, Ministry of Energy and Infrastructure, Abu Dhabi 11191, United Arab Emirates; 4 Department of Chemistry, College of Arts and Sciences, University of Petra, Amman 11196, Jordan; 5 Department of Chemistry, The University of Jordan, Amman 11942, Jordan

**Keywords:** galena, PbS, high-vibration milling machine, PbS nanoparticles

## Abstract

In this research, some physical properties such as the density, specific heat capacity, and micro-hardness of galena ore lumps purchased from the public market were determined. The microscopic study, using the scanning electron microscopy (SEM) and energy dispersive X-ray spectroscopy (EDS), confirmed that the as-received galena ore was mostly lead sulfide (PbS). The XRD pattern of the galena powder also elucidated that all the peaks were assigned to PbS. In addition, the as-received galena was roughly crushed, and fine-milled using a high-vibration milling machine with tungsten carbide rings. Nanoscale particles of about 90 nm were produced in a very short milling time of around 15 min. The obtained nanoscale powder was well investigated in the SEM at low and high magnifications to assess the exact range of particle size. Meanwhile, the SEM was employed to investigate the microstructure of sintered samples, where a part of the milled galena powder was compacted and sintered at 700 °C for 2 h. Again, the result of this investigation proved the formation of PbS with even smaller grain size compared with the grain size of the starting galena ore. A high relative sinter density of approximately 97% for galena powder was achieved by sintering under vacuum.

## 1. Introduction

Galena is the main source of lead extraction, which is widely used in industrial manufacturing, including batteries. Galena mainly consists of lead sulfide (PbS) and other trace elements such as silver, zinc, silicon, iron, copper, and their oxides [1,2]. Depending on the mining site, many types of galena with different chemical composition have been reported [3], and it has been confirmed that the richest lead and sulfur galena ores were found in the USA and Japan. As long as galena mineral is the main source of lead, it will remain the main source of PbS compounds for many applications, such as thermoelectric parts, infrared detectors, solar selective surfaces, semiconductors, and solar cells [4,5,6,7].

Nanotechnology, in the subject of PbS applications, has had a very effective role in improving and developing the properties of this compound for various applications. The physical and chemical properties of nanomaterials depend on the starting raw materials, production conditions, particle size range of the obtained nanoparticles, and the subsequent heat treatment processes. Most of nanomaterials’ applications in this field are in the form of thin films. This subject has been well covered by many workers in this advanced field, to investigate the synthesis methods and applications of nano-lead sulfide compounds [8,9,10,11,12].

On the other hand, the powder metallurgy technique is particularly used to produce macro, micro, ultrafine, and nanoparticles for different applications as required, starting from bulk compounds or alloys. This technique is typically used to produce alloys or compounds that cannot be manufactured by the traditional casting method, or to improve the properties of any product by controlling its chemical composition and microscopic features up to semi-shape production stage. Obviously, the physical, chemical, and mechanical properties of powder technology products depend on the starting powder characteristics, particle size distribution range, compaction pressure, sintering temperature, post sintering course, and sintering atmosphere. All these factors play a vital role in governing the final properties of the product. Significant information about powder metallurgy and its applications, and where it must be used in the production process, has been reported by many researchers [13,14,15,16,17,18,19,20]. A recent review by Joy et al. summarized some previous work on the mechanochemical milling processes [21]. Based upon the above information about powder metallurgy, galena ore can be used to produce ultrafine or nanoparticle powder. There is a lack of information concerning the subject of production of fine powder of PbS starting from galena ore, which already differs from one place of mining to another. Meanwhile, the chemical composition, physical properties, and its microstructure differ as well.

Thus, the novelty of the present research is to achieve the following objectives:

(i) To investigate the microstructure and chemical composition of as-received galena using scanning electron microscopy in conjugation with its energy dispersive X-ray spectrometer, as the properties of each batch of galena ore depend on its mining place.
(ii) To determine, as a function of temperature, some physical properties for the used galena, such as density, specific heat capacity, and hardness.
(iii) To produce ultra-fine and nanoscale particles of lead sulfide (PbS) starting from a commercial galena ore by employing high-vibration mechanical milling.

## 2. Experimental

### 2.1. Materials and Methods

- The following materials and instruments were used for conducting the practical work:
- Lumps of galena ore were purchased from a local market in Amman, Jordan.
- Digital micro-balance (Model SEJ-205, Taipei, Taiwan).
- Digital caliper with minimum reading 0.01 mm (Total, TMT 322001, Guangzhou, China).
- Scanning Electron Microscope (SEM), with integrated Energy Dispersive X-ray Spectrometer (EDS) (thermoscientific, JU-24112022, Waltham, USA).
- SEM (Inspect F50-FEI Company, Eindhoven, Netherlands).
- Differential Scanning Calorimeter (DSC) (NETZSCH DSC 204 F1).
- X-ray diffraction system (XRD) (Philips PW-1710), 40 kV and 30 mA with a Cu tube operating.
- Vibrating milling machine (TEMA, Woodford Halse UK) including tungsten carbide multi-rings with solid tungsten core.
- Micro Vickers Hardness Tester (MVHS), Mod/HTMV 2000M, SN/20190000187, Italian made, echo LAB.
- Different grades of emery papers for grinding and three types of diamond pastes for surface polishing.
- Compaction press, type Carver Lab. Press, model C-31000-564, UK.
- Homemade stainless-steel punch-die compaction system with a diameter of 1.21 cm.
- Vacuum oven (JEIO TECH, MODEL OV-11, AAH13115K, Korean made), temperature range from room temperature to 300 °C.

Digital thermometer (JR-1, China), −50–300 °C, accuracy ± 0.1 °C.

### 2.2. Investigation of the Physical Properties of Used Galena Ore

Some physical properties of the as-received galena were investigated, such as density, specific heat capacity, and micro-hardness.

#### 2.2.1. Density Determination

Lumps of galena ore were purchased from the local market in Amman. The density of the as-received galena lumps was measured by water displacement and geometrical measurement methods. Different size pieces were weighed using the digital micro-balance and then they were submerged in a graduated glass tube containing distilled water of known volume. Thereby, the volume of the galena pieces was found through the volume difference after submerging the pieces in water, and then the density was calculated from the law of density (ρ = mass/volume). The density of a galena lump was also determined by its geometrical dimension measurements and the mass of the lump. The selected lump was carefully ground to get a regular cubic shape, and its dimensions were measured by the digital caliper. It was necessary to measure the density of the as-received galena for the purpose of comparison with the reported density, and with green and sinter densities after the production of galena bulk by powder metallurgy method, as will be seen later.

#### 2.2.2. Specific Heat Capacity Determination Using Calorimetry and Differential Scanning Calorimeter (DSC) Techniques

The specific heat capacity of the as-received galena bulk was calculated using a well-insulated calorimeter to find the specific heat capacity for bulk metals, as an approximate method. The following basic equations can be used to determine the specific heat capacity of the as-received galena lumps using the calorimetry technique [22]:

(1) $$q_{cold}=-q_{hot}$$

(2) $$m_{w\;}c_{w}\;\left(T_{f}-T_{w}\right)=-m_{g\;}c_{g}\;\left(T_{f}-T_{g}\right)$$

where *q_cold_* is the heat gained by water, *q_hot_* is the heat lost by the unknown heated sample, *m_w_* and *m_x_* are the masses of the water and the galena sample, and *c_w_* and c_g_ are the specific heat capacities of water and the galena sample, respectively. *T_w_* and *T_g_* are the initial temperatures of water and the galena sample, respectively, and *T_f_* is the system equilibrium temperature. Therefore, Equation (2) can be projected and solved to find the specific heat capacity of the galena ore *c_g_* as follows:

(3) $$c_{g}=\frac{m_{w\;}c_{w}\;\left(T_{f}-T_{w}\right)}{m_{g\;}\;\left(T_{g}-T_{f}\right)}$$

The masses of the used galena and water were weighed by a digital micro-balance. The initial water temperature was measured using the digital thermometer, and the initial temperature of the galena bulk sample was also measured after heating in a water bath. The heated galena was submerged in the calorimeter, then agitated a little for good heat exchange, and then the maximum equilibrium temperature was measured. The specific heat of the tested galena was calculated using Equation (3). The test was repeated three times for reasonable accuracy while the result of one run was tabulated.

The DSC test was conducted to accurately find the specific heat capacity at constant pressure (*c_p_*). Lumps of galena ore were roughly crushed to about 0.3 mm particle size to run the DSC test from room temperature to ~150 °C, for assessing the value of *c_p_* of the ore with respect to the applied temperature range. The curves of the heat flow over the tested sample mass (mW/mg) as a function of temperature were drawn. The *c_p_* of the as-received galena in the test temperature range was calculated and drawn according to Equation (4) [23].

(4) $$c_{p}=\frac{HF}{m\;\beta }$$

where *c_p_* is the specific heat capacity at constant pressure, *HF* is the heat flow, *m* is the mass of the sample, and *β* is the applied heating rate. The final SI unit of the above equation for *c_p_* is J/kg·K.

#### 2.2.3. Vickers Micro-Hardness Measurement

Micro-hardness of the as-received galena ore was measured using the MVHS. The tested specimen of a total surface area of approximately 2.5 cm^2^ was ground using emery papers with a wide range grade (220 to 2000). The specimen must be rotated by 90° when switched from one stage of grinding to another in sequence, and washed well with water between each stage to remove any residual particles or contamination. The surface was then polished with diamond paste of 7 µm, 2.5 µm, and 1 µm, in order. Between each stage of polishing, the specimen was washed with alcohol, and dried with soft tissue paper. The micro-hardness test was repeated 10 times using 0.98 N force, and the mean value was then reported.

### 2.3. Scanning Electron Microscopy (SEM) Characterization of the As-Received Galena Ore

SEM was used to examine the starting lumps of galena. A fractured surface was well chosen to be almost planar for a good features test. The prepared specimen was carefully washed with distilled water, alcohol, and dried to be ready for the SEM test, after fixing it on an aluminum stub using a double adhesive carbon sticker. Meanwhile, the EDS that is integrated with the SEM was used for chemical analysis. The milled powder was also examined to explore the particle size range and the shape of particles, in general. In addition, some specimens of sintered powder were examined for microstructural and chemical investigations.

### 2.4. Milling Process of Galena Ore

Some lumps of galena were crushed into small pieces of approximately 2 mm or less in diameter using a steel mortar. After that, the product was dry-milled using the tungsten carbide multi-rings with a solid core tungsten carbide milling machine (TEMA). The milling vessel and the tungsten carbide parts were cleaned well using an ultrasonic cleaner before milling. The milling parameters are as follows: time, 15 min.; medium, air atmosphere; vibration frequency, 3000 min^−1^. Some of the produced powder was submerged in distilled water for 5 min and decanted to separate the fine powder. The stilled down powder was then rinsed with ethanol (96%) and dried under vacuum at about 80 °C for 15 min using the vacuum oven. The SEM was used to examine the produced powder to identify the expected particle size and shape to support the results of this research.

### 2.5. X-ray Diffraction (XRD) Test

The XRD technique was used to recognize the phases of the as-received galena ore. A sample of galena powder was tested in the range of 5° to 60° of 2 θ with a 2 s time interval. The obtained XRD pattern was analyzed and labeled accordingly.

### 2.6. Compaction and Sintering of Galena Powder

Two samples of galena powder were compacted using the homemade compaction system with a pressure of 300 MPa. They are the milled powder of 15 min milling time and the water-separated powder. The obtained compacted samples were then sintered in vacuum of about 10^−3^ torr at a temperature of 700 °C for 2 h with a heating rate of 10 °C/min. The rate of cooling to room temperature was the same rate of heating while the sample stayed under vacuum. The densities at room temperature for the green and sintered products were calculated for comparison with the density of the as-received galena, using the same method explained in Section 3.1.

## 3. Results and Discussion

### 3.1. Density Determination

**Figure 1:**
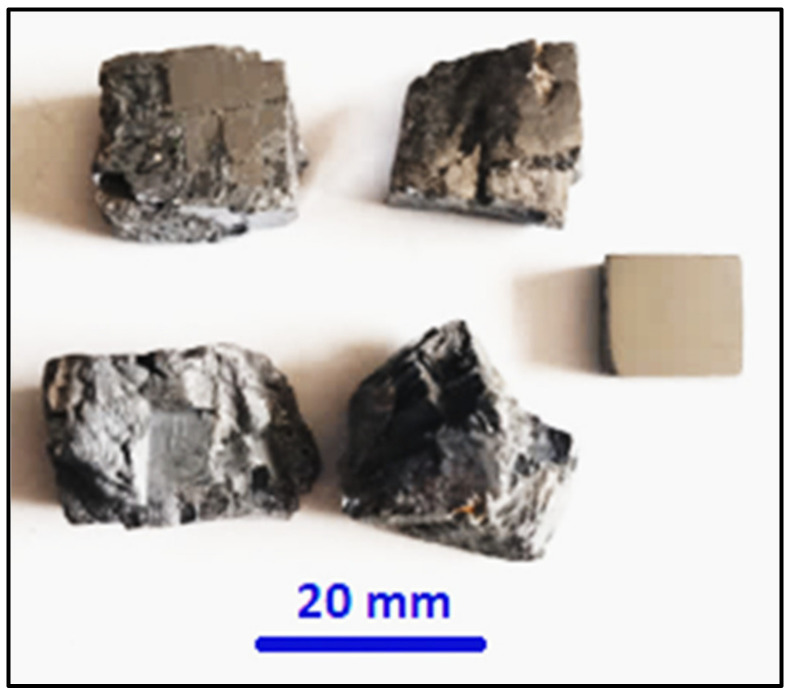
Some raw lumps of galena with one ground cubic shape.

Our results for the density measurement of the as-received galena ore using two applied methods were very close in value. The first method was performed by measuring the dimensions of a well-cut cube of galena in order to determine its volume. Mass is then divided by the volume to calculate the density. The second method employed the water displacement technique to find the volume of the ore. The mass and volume were then used to calculate the densities. Density values were approximately 7.35 × 10^3^ kg/m^3^ and 7.29 × 10^3^ kg/m^3^ for the two methods, respectively, which are less than the published value (7.55 × 10^3^ kg/m^3^) [24]. Generally, the mismatch of physical or chemical properties of galena ore is due to the different mining locations in the world. Figure 1 shows some lumps of galena and the ground cubic one that were used for density measurement.

### 3.2. Specific Heat Capacity Measurement Using Calorimetri Cand DSC Data

The specific heat capacity of the as-received galena was determined using the two mentioned routes: calorimetry and DSC methods. Table 1 summarizes the data of one run of the calorimeteric method. The obtained approximate value of specific heat capacity was found to be 215 J/kg·°C which is very close to the published value of 210 J/kg·°C [24].

**Figure 2:**
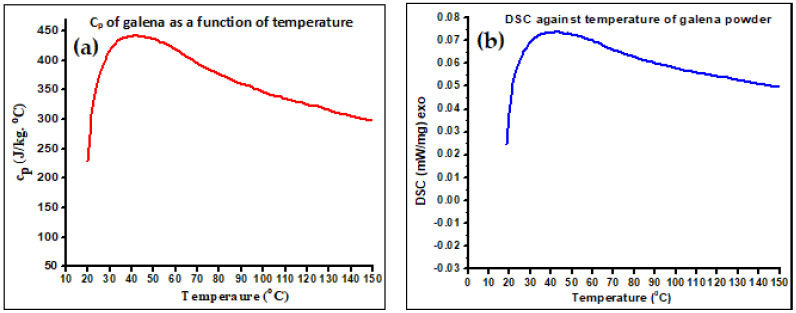
(**a**) The c_p_ curve of the as-received galena ore against temperature. (**b**) DSC, heat flow per unit mass with temperature.

In addition, the specific heat capacity of the as-received galena in the range from room temperature to around 150 °C was considered using the DSC system to assess the variation of c_p_ as a function of temperature. The resulting curves are illustrated in Figure 2, after converting the DSC data (mW, mg, and min) into SI units and applying Equation (4) to calculate the specific heat capacities of the taken temperature range. The value of c_p_ that was found using the calorimeter method at room temperature is very close to the DSC technique value at room temperature. The curve of the c_p_ increases sharply from room temperature up to about 35 °C, and then starts descending up to the end experiment. To our knowledge, we believe that this curve for c_p_ as a function of temperature for the galena ore in this range of temperature is reported for the first time. In 2022, the specific heat capacity of PbS (galena) in the range of 12.20 K to 338.22 K was measured [25]. The reported values of c_p_ are lower than ours for the same temperature range in our DSC measurement (18–150 °C ~ 291–423 K) (see Figure 2a). For comparison purposes, the literature’s result for the c_p_ of PbS (galena) was converted from J/mol.K into J/kg·K (the molar mass of pure PbS is 239.27 g/mol). For example, the value of c_p_ that was calculated from our DSC data at about 20 °C was 228 J/kg·°C while their result at the same temperatures was 210 J/kg·°C. This variation in the specific heat capacity values of galena ores can be explained on the basis of the difference in their chemical composition according to the mining site.

### 3.3. Micro-Hardness Measurement

Table 2 contains 10 values of micro-hardness measured for the as-received galena ore polished surface. The mean value of the Vickers micro-hardness test was approximately 77.25 HV, which represents the general value for the used galena. This value is very close to the range of hardness published in the literature [26].

As a summary, we had to investigate some physical and chemical properties of the used galena since they mostly differ according to the place of mining. The determined physical properties of the as-received galena are summarized in Table 3.

### 3.4. Surface Characterization of the As-Received Fractured Galena Ore

**Figure 3:**
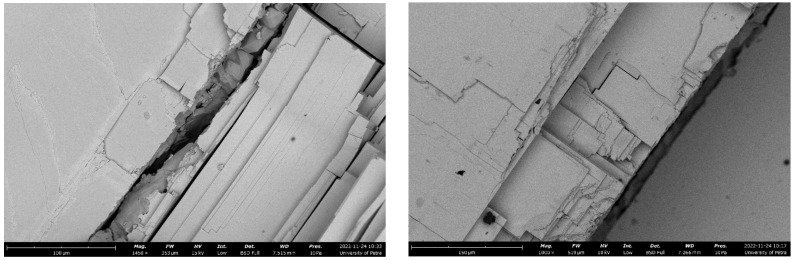
SEM micrographs (BSI) for different fracture surfaces of the used galena. Relevant parametes for the image on the left: scale bar: 100 um, mag: 1450×, HV: 25 kV, Date: 24 November 2022. Relevant parametes for the image on the right: scale bar: 150 um, mag: 1000×, HV: 10 kV, Date: 24 November 2022.

**Figure 4:**
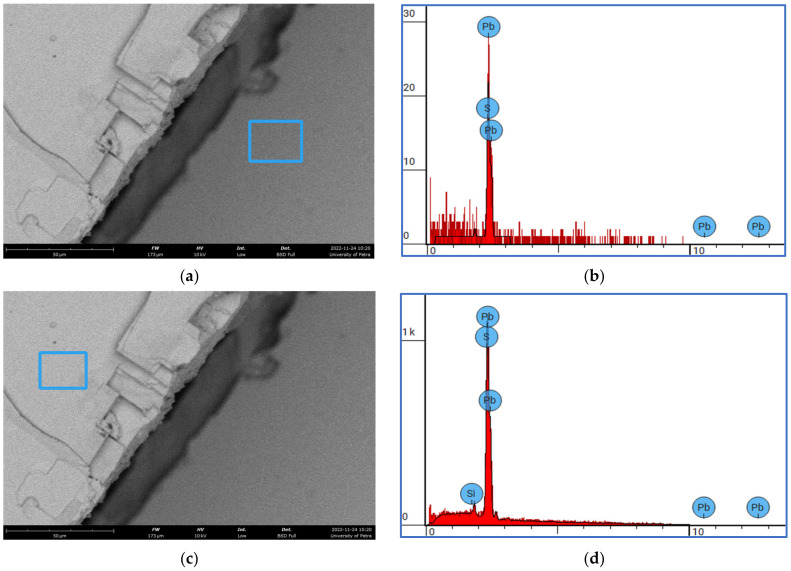
(**a**) SEM micrograph (BSI), fractured surface of the as-received galena, (**b**) the EDS spectrum for the blue square in (**a**), (**c**) the other side of the fractured surface, (**d**) the EDS spectrum of the blue square in (**d**).

The SEM micrographs and the EDS spectrum were used to investigate the microstructural and chemical analysis of the examined fractured surface of the used galena. Figure 3 shows the features of the main phase of PbS in the as-received galena ore. Here, we can see the expected 90 degrees fracture cleavage. Figure 4 illustrates two different areas of the fractured surface, that is dominantly PbS, together with their EDS spectrums. The chemical analysis confirmed that the PbS main phase in the used galena was approximately 85% by mass of Pb and 14% by mass of S. Table 4 includes the chemical composition of the analyzed blue square areas in Figure 4.

### 3.5. Milling of Galena Ore

The milling time is considered very short when compared with many other milling processes such as the ball milling techniques. However, we were able to produce ultra or nanoscale particle sizes because of the very fast vibration of the milling machine, as well as because the tungsten carbide rings and the core part are extremely hard.

**Figure 5:**
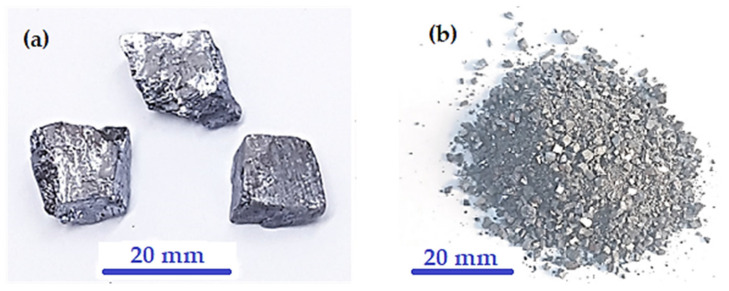
(**a**) The as-received starting galena lumps. (**b**) Rough pulverized galena product.

**Figure 6:**
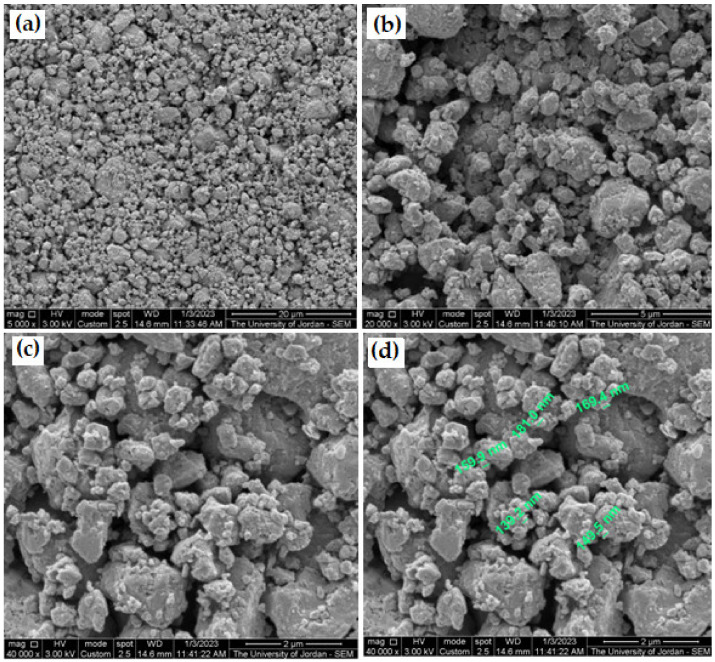
SEM micrographs (SEI) of galena powder after 15 min milling time using different magnifications. (**a**) 5000×, (**b**) 20,000×, (**c**) 40,000×, and (**d**) same micrograph of (**c**) with nanoscale labeling.

**Figure 7:**
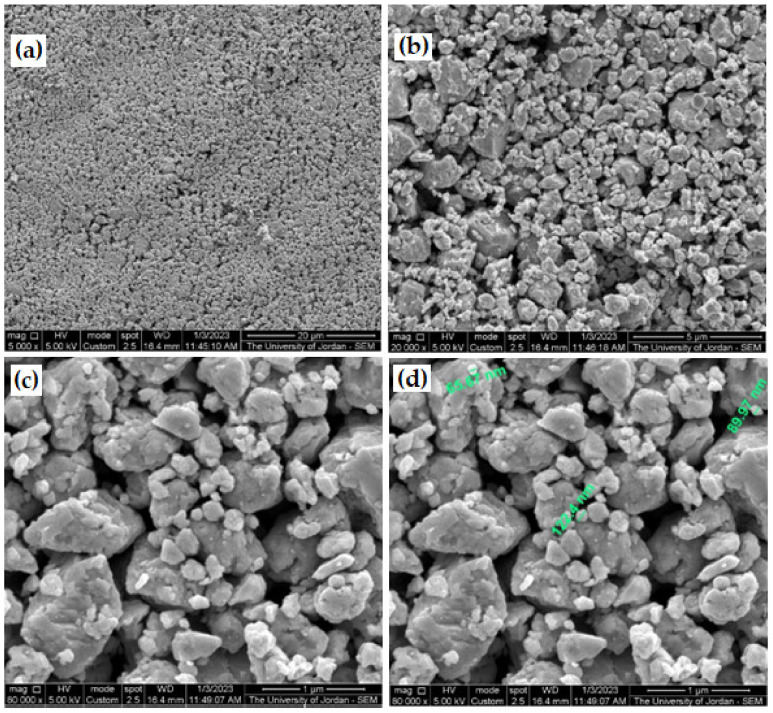
SEM micrographs (SEI) of water-separated galena powder for 5 min using different magnifications. (**a**) 5000×, (**b**) 20,000×, (**c**) 80,000×, and (**d**) same micrograph of (**c**) with nanoscale labeling.

Figure 5 shows photographs of three as-received galena lumps together with some pulverized galena prior to the fine milling stage. The fine produced powder, macro, and nanoscale particles size are shown in Figure 6. It is clear from the figure that the used milling machine can be employed to produce ultra-fine powder of brittle alloys or compounds, such as the galena ore. The reason for finding ultra-fine powder from galena is due to the very high brittleness of its texture. As for the powder resulting from the employed milling process for 15 min, the SEM examination confirmed reaching the nanoparticles scale, as shown in Figure 6d. In addition, the SEM micrographs of galena powder separated by water for 5 min showed less particle size range, as illustrated in Figure 7.

**Figure 8:**
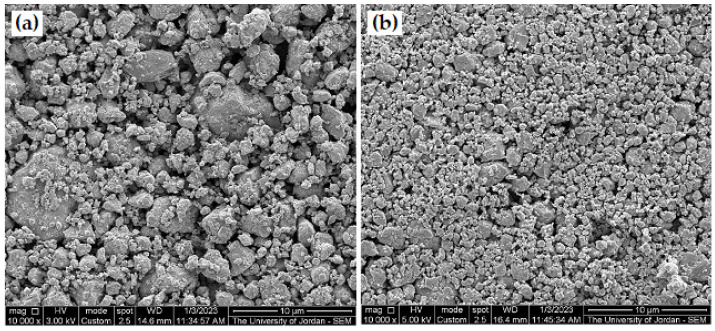
SEM micrographs (SEI) at same magnification 10,000× (**a**) general image, as milled galena powder after 15 milling time, (**b**) image of water separated galena powder from the produced powder of 15 min milling time.

For comparison between milled galena powder of 15 min milling time and water-separated powder, two images of SEM with the same magnification are shown in Figure 8. From which, we can recognize the noticeable difference in particle size range after powder water separation, as it is reduced by about 50%. Therefore, this way of powder separation can be adopted to produce ultra-fine powder from the milled powder that is not reactive with water. If the produced powder is affected when submerge in water, water can be replaced with any inert solvent/solution for the separation. Meanwhile, the particle size range depends on submerging time, densities of the used liquid, and the separated powder material.

The general conclusion from the above is that this milling technique we employed can be effectively used to produce ultra-fine powder down to the nanoscale particle size in a short time period (approximately 15 min), and it depends on the milling time. Additionally, water can be used to separate powders with different particle sizes, for materials that are not reactive with water, to obtain very fine powder required for many applications.

### 3.6. X-ray Diffraction Test

**Figure 9:**
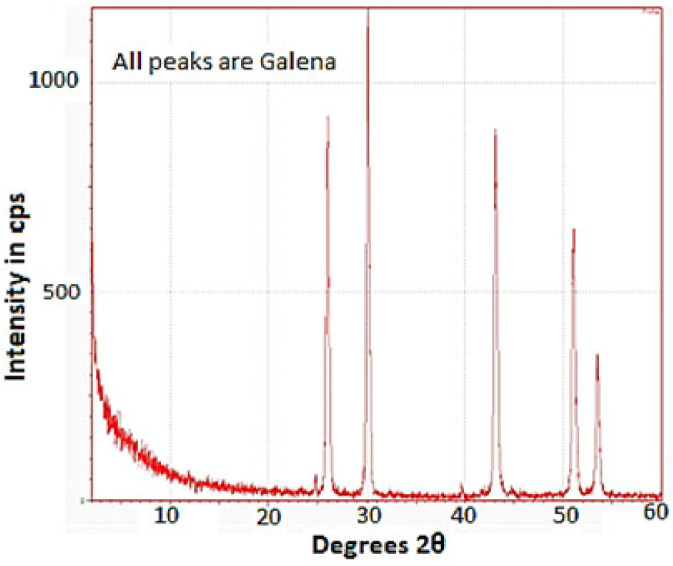
The XRD pattern of the as-received galena powder.

The obtained XRD pattern of the as-received galena powder using the XRD technique confirms that all peaks of the XRD curve are attributed to pure PbS as a main phase, as seen in Figure 9. The curve also does not show any oxygen peaks, which means there is no oxidation during the milling of galena ore under atmospheric conditions. This new finding is very important from a production standpoint to prepare PbS powder starting from galena ore in air for different applications.

### 3.7. Yield of Compaction and Sintering of Galena Powder

The green densities of compacted and sintered samples were calculated from geometrical dimensions and their masses for 15 min milled powders and water-separated ones as summarized in Table 5. The green densities of the three cases recorded in the table are very close to each other. The reason for this result probably is due to the wide range distribution of the particle size of the produced powder in all cases. The relative sintered densities (*ρ_rel_*._%_) are approximately 97% of the measured bulk density of the as-received galena ore, and is recorded in Table 3. This percentage can be calculated as follows:

(5) $$\rho_{rel.\%}=\frac{\rho_{Sint.}}{\rho_{Bulk}}\times 100=\frac{7.11\times 10^{3}\;\mathrm{kg}/m^{3}}{7.32\times 10^{3}\;\mathrm{kg}/m^{3}}\times 100\approx 97\%$$

This value is very encouraging for the production of PbS (galena) in various forms of practical applications, using the powder technology route. Mostly, the relative density can be augmented to approach the reference density when the compaction pressure is high enough to get products of high green density, and when the sintering process is carried out under high vacuum with suitable sintering temperate and time.

**Figure 10:**
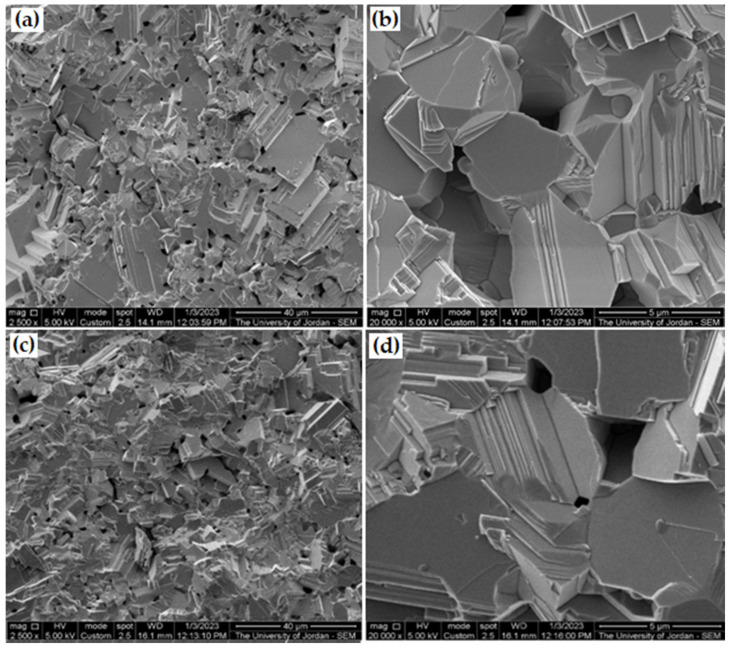
SEM micrographs (SEI) of sintered samples’ fractured surfaces: (**a**) powder of galena of 15 min milling time at 2500×, (**b**) same of (**a**) at 20,000×, (**c**) sample of water separated galena powder at 2500×, and (**d**) same of (**c**) at 20,000×.

The sintering process must be carried out under vacuum or inert gas to avoid oxidation, unless otherwise is required. The fractured surface images of sintered samples using the SEM confirm reaching high sintered density products. Figure 10 illustrates many SEM images with low and high magnifications of sintered samples’ fractured surfaces of 15 min milled powder, which is characterized by low void density. The figure shows the same fractured surface feature in comparison with the as-received galena shown in Figure 3, but with a smaller grain size. Additionally, it clearly confirms the formation of pure PbS phase as the main phase again, after the vacuum sintering process, starting from the galena powder.

**Figure 11:**
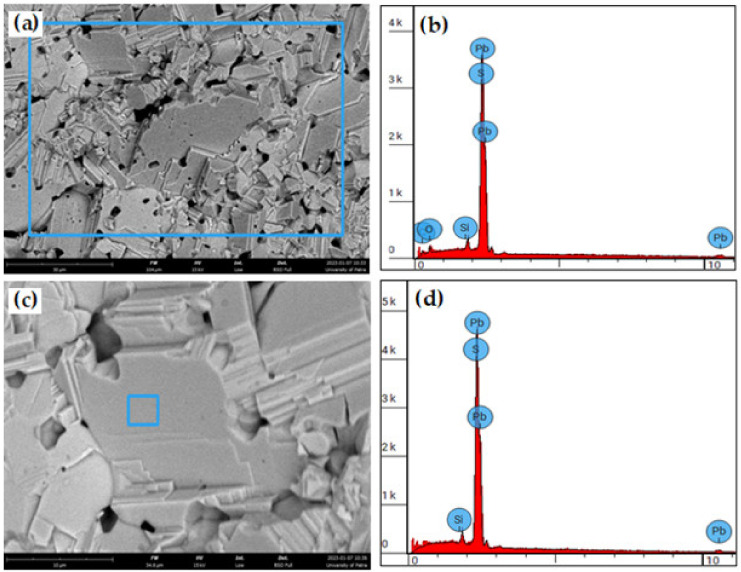
SEM micrographs (BSI) with their EDS spectrums of sintered galena powder: (**a**) fractured surfaces, magnification scale 30 µm, (**b**) EDS spectrum for (**a**), (**d**) higher magnification, PbS phase, and (**d**) EDS spectrum for (**c**). Blue squares represent the sampled area for EDS.

The EDS unit that is integrated with the thermoscientific SEM gave some more details about sintered samples of galena. Figure 11 shows two SEM micrographs with low (a) and high (c) magnifications, together with their ESD spectrums (b) and (d), respectively. The chemical compositions of the two blue squared areas are given in Table 6. The EDS test confirmed the formation the PbS main phase after the sintering process of galena powder, and gave the qualitative and quantitative chemical composition.

## 4. Conclusions

The novel findings of the current research can be summarized as follows:

- Some physical properties of the as-received galena lumps have been determined, such as density, specific heat capacity, and micro-hardness, as they depend on the mining site.
- The microstructures, chemical analysis of the as-received galena, the produced powder, and sintered samples were investigated using the SEM microscopy. The result confirmed that the main phase of the used galena was pure PbS.
- We have succeeded in producing nanoparticles of approximately 90 nm and ultra-fine powder of PbS, using a simple high-vibration tungsten carbide ring milling machine in a very short time of approximately 15 min under atmospheric conditions.
- The sintering process of galena powder must be carried out in vacuum or under inert gas to avoid oxidation.
- A relative density of approximately 97% of sintered galena produced powder was achieved at the sintering temperature of 700 °C for 2 h, which is a very encouraging result for practical applications.
- The milling and sintering of high PbS phase percentage galena ore can be employed to produce the bulk of PbS for different scientific applications as required.

## Figures and Tables

**Table 1:** Real data of the specific heat capacity experiment for the galena ore.

Parameters	Galena	Water
m_g_ and m_w_ (kg)	0.06332	0.08044
c_g_ and c_w_ (J/kg·°C)	x (unknown) ^1^	4186
T_g_ and T_w_ (°C)	87.1	17.8
T_f_	20.6	20.6

^1^ x = c_g_ = 214.86 J/kg·°C by using Equation (3).

**Table 2:** Measured values of micro-hardness of polished sample of galena lump.

Sample No.	Micro HV	Used Load
1	81.33	0.98 N
2	80.28	
3	80.07	
4	78.43	
5	78.56	
6	69.75	
7	71.82	
8	72.53	
9	79.23	
10	80.54	
Mean value	77.25	

**Table 3:** Physical properties of the as-received galena ore.

Property	Value in the SI Units
Density	7.32 × 10^3^ kg/m^3^
Vickers Hardness	77.25 HV
Specific heat capacity	214.86 J/kg·K

**Table 4:** Chemical analysis of the used galena, employing the EDS unit of the SEM.

Tested Area	Element Atomic Number	Element Symbol	Element Name	Atomic %	Mass %
Figure 4b	16	S	Sulfur	55.17	16.00
	82	Pb	Lead	44.83	84.00
Figure 4d	14	Si	Silicon	0.83	0.20
	16	S	Sulfur	50.95	14.03
	82	Pb	Lead	48.22	85.77

**Table 5:** Full description of compaction and sintering process of the used galena ore.

Powder Description	Compact Density ×10^3^ kg/m^3^	Sintered Density ×10^3^ kg/m^3^	Compaction Pressure MPa	Sintering Condition
Powder after 15 min. milling time	6.00	7.11	300	700 °C, 2 h, rate of heating 10 °C/min, under vacuum of about 10^−3^ torr, furnace cooling at the same rate of heating
Powder after suspension in water, and separated	5.95	7.13		
Residual powder after removing the fine powder of water suspension	6.08	7.04		

**Table 6:** Chemical composition of sintered sample of galena powder.

Analyzed Area	Element Name	Element Symbol	Atomic Conc.	Mass Conc.
Figure 10a Blue square	Carbon	C	17.08	2.50
	Oxygen	O	13.68	2.60
	Silicon	Si	0.87	0.21
	Sulfur	S	35.26	13.40
	Lead	Pb	33.10	81.28
Figure 10c	Sulfur	S	50.95	14.03
Small Blue square	Silicon	Si	0.83	0.20
	Lead	Pb	48.22	85.77
